# NF90 Binds the Dengue Virus RNA 3′ Terminus and Is a Positive Regulator of Dengue Virus Replication

**DOI:** 10.1371/journal.pone.0016687

**Published:** 2011-02-28

**Authors:** Raúl C. Gomila, Glover W. Martin, Lee Gehrke

**Affiliations:** Division of Health Sciences and Technology and Department of Microbiology and Molecular Genetics, Harvard Medical School, Boston, Massachusetts, United States of America; George Mason University, United States of America

## Abstract

**Background:**

Viral RNA translation and replication are regulated by sequence and structural elements in the 5′ and 3′ untranslated regions (UTR) and by host cell and/or viral proteins that bind them. Dengue virus has a single-stranded RNA genome with positive polarity, a 5′ m7GpppG cap, and a conserved 3′-terminal stem loop (SL) that is linked to proposed functions in viral RNA transcription and translation. Mechanisms explaining the contributions of host proteins to viral RNA translation and replication are poorly defined, yet understanding host protein-viral RNA interactions may identify new targets for therapeutic intervention. This study was directed at identifying functionally significant host proteins that bind the conserved dengue virus RNA 3′ terminus.

**Methodology/Principal Findings:**

Proteins eluted from a dengue 3′ SL RNA affinity column at increasing ionic strength included two with double-strand RNA binding motifs (NF90/DRBP76 and DEAH box polypeptide 9/RNA helicase A (RHA)), in addition to NF45, which forms a heterodimer with NF90. Although detectable NF90 and RHA proteins localized to the nucleus of uninfected cells, immunofluorescence revealed cytoplasmic NF90 in dengue virus-infected cells, leading us to hypothesize that NF90 has a functional role(s) in dengue infections. Cells depleted of NF90 were used to quantify viral RNA transcript levels and production of infectious dengue virus. NF90 depletion was accompanied by a 50%-70% decrease in dengue RNA levels and in production of infectious viral progeny.

**Conclusions/Significance:**

The results indicate that NF90 interacts with the 3′ SL structure of the dengue RNA and is a positive regulator of dengue virus replication. NF90 depletion diminished the production of infectious dengue virus by more than 50%, which may have important significance for identifying therapeutic targets to limit a virus that threatens more than a billion people worldwide.

## Introduction

Dengue virus is a member of the family *Flaviviridae,* which comprises single stranded positive sense RNA viruses such as West Nile Virus (WNV), Japanese encephalitis virus (JEV), yellow fever (YF) virus, as well as the pestivirus bovine viral diarrhea virus (BVDV) and the hepacivirus, hepatitis C virus (HCV). Dengue virus infections are a significant global health concern. Approximately 100 million cases of dengue fever infections (DF) are reported annually, of which 250,000–500,000 cases comprise the more severe and life-threatening dengue hemorrhagic fever (DHF) [1]. It is estimated that 2.5 billion people live in areas that are at risk for dengue outbreaks [2], mainly tropical and subtropical areas that are coupled to the distribution of the virus' biological vectors: *Aedes aegypti* and *A. albopictus* mosquitoes. There are four dengue serotypes, and DHF is linked to sequential infection by mosquitoes carrying different serotypes [1]. This effect, termed antibody dependent enhancement (ADE), is thought to occur by the presence of non-neutralizing antibodies that facilitate the infection and increase virus titer [3].

Flavivirus genomic RNAs do not have a 3′-terminal poly(A) tract; rather, the viral RNAs have a 3′ UTR (400–700 nucleotides in length) that is predicted to form significant secondary structure, with a stable terminal 3′ stem loop structure (3′ SL). This structure was first proposed by Grange *et al*. [4] by analyzing the cDNA sequence of the YF virus 17D vaccine strain. Brinton *et al*. [5] proposed that, although there was primary sequence divergence, the overall 3′SL structure was highly conserved among WNV, St. Louis encephalitis virus (SLE) and YF viruses. The structural conservation of this element suggests that it could have a common function in the life cycle of flaviviruses. Indeed, during replication of positive strand RNA viruses, sequences and structures within the 3′UTR serve as the promoter for minus strand synthesis. The 3′SL was identified as a key regulatory element for the *in vitro* synthesis of minus strand dengue virus RNA [6]. The putative flavivirus replicase complex (NS3/NS5) was shown to bind the 3′UTR only when the 3′SL was present [7]. Mutational analysis conducted by Zeng *et al*. revealed that the dengue virus 3′SL contains structural and sequence elements that are required for replication of the virus [8]. Results from an *in vitro* polymerase assay by You *et al*. supported the conclusion that the structure, not the sequence of the top half of the 3′SL, is important for replication [6]. The authors also found that disrupting the pseudoknot structure affected the *in vitro* transcription activity of the RNA-dependent RNA polymerase (RdRp). Similarly, Bredenbeek *et al*. did not detect viral RNA replication after deleting the 3′SL of a yellow fever construct [9]. Moreover, recent studies show that nucleotide substitutions [10], [11], as well as the location of bulged nucleotides [12] along the long stem loop structure affect WNV replication. Together, these data underscore the importance of the flavivirus 3′SL in the life cycle of the flaviviruses.

The conserved flavivirus 3′ SL may also regulate viral RNA translation. Holden and Harris showed that deleting the 3′SL reduced the translation of reporter constructs that included the dengue virus RNA 5′ and 3′UTR regions [13]. The positive effects of the 3′SL required the 5′ m7GpppG cap structure. In a separate study, Holden *et al.* showed that specific targeting of the top loop in the 3′SL structure of dengue virus RNA using peptide-conjugated phosphorodiamidate morpholino oligomers (P-PMOs) inhibited translation and replication [14]. However, other studies suggest that the 3′SL is not involved in enhancing translation or may actually inhibit it. Tilgner *et al*. reported that deleting the 3′SL, and of most of the 3′UTR, of a WNV replicon containing a luciferase reporter, had no effect on reporter activity during the early time points post electroporation [10], when translation is being measured. Li and Brinton proposed that the WNV RNA 3′SL inhibits reporter construct translation [15], possibly by sequestering essential host translation factors.

To identify host proteins with potential to regulate viral RNA replication and translation, we applied a combination of biochemical methods and functional assays. RNA affinity chromatography identified several proteins that eluted with increased ionic strength, including NF90 and RHA, members of the double stranded RNA binding protein family (dsRBP), along with NF45, the binding partner of NF90. Although NF90 and RHA localized to the nucleus in uninfected cells, cytoplasmic NF90 was also detected by immunofluorescence imaging in the cytoplasm of dengue virus-infected cells, thereby directing us to focus on the potential functional significance of NF90 in the dengue life cycle. Human melanoma cells that were depleted of NF90 by constitutive expression of an NF90 shRNA were used to further examine the functional significance of NF90 in dengue virus-infected cells. NF90 depletion was accompanied by a 30%–50% decrease in dengue virus RNA accumulation, and up to a 70% decrease in infectious virus production. Coupled with experimental analyses of related viruses by other investigators [16], [17] these results are evidence that NF90, RHA, and NF45 are isolated in complex with the dengue virus 3′ SL RNA, and that NF90 is a positive regulator of dengue virus production.

## Results

### Purification of dengue 3′SL RNA binding proteins

**Figure 1:**
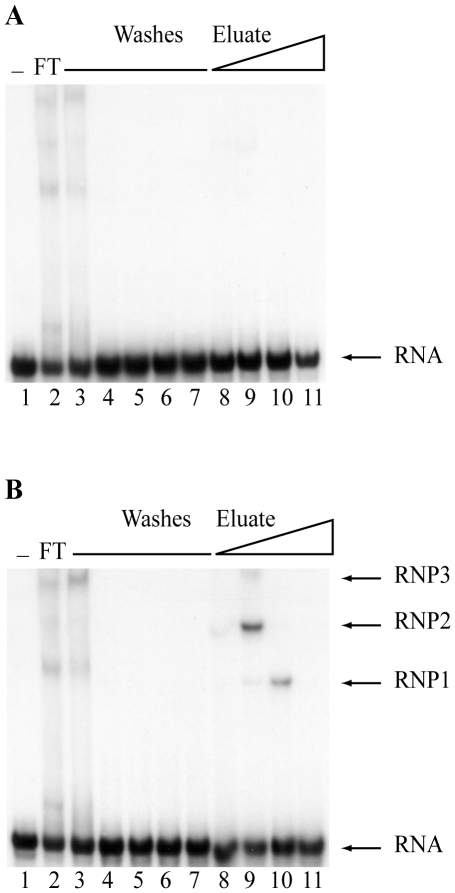
RNA binding activities eluted from the dengue 3′SL RNA columns. S10 extract from K562 cells was prepared and chromatographed as described in the Materials and Methods section. In both panels, lane 1 represents the dengue 3′SL RNA only. The second lane shows the binding properties of proteins that washed through the column in the flow through (FT) fraction. The five column washes are analyzed in lanes 3–7. Bound proteins were step-eluted with 250 mM, 500 mM, 1 M and 2 M NaCl (lanes 8–11 respectively). (A) The S10 extract was pre-cleared by passing it over the control (lacking bound RNA) column. (B) The pre-cleared extract was chromatographed on the dengue 3′SL affinity column. The EMSA using the fractions eluted from the RNA affinity column shows three binding activities eluted from the column, RNP1, RNP2, and RNP3 (lanes 9–11).

We used RNA affinity column chromatography [18], [19] to identify proteins in complex with the dengue 3′SL RNA (Figure 1). After passing pre-cleared cell extracts over the RNA-coupled affinity column, a stepwise elution was performed using buffers containing increasing NaCl concentrations to distinguish low affinity from higher affinity interactors. RNA binding activity in the fractions was assessed by electrophoretic mobility shift assay (EMSA). For the control column (matrix only), RNA-protein complexes were detected only in the flow-through or initial wash fractions (Figure 1A, lanes 2 and 3). The absence of any significant shifted bands in further washes or NaCl elution fractions (Figure 1A, lanes 3–11) indicates that the matrix has minimal non-specific binding activity.

The corresponding results with the dengue 3′ SL RNA-coupled affinity column (Figure 1B) showed similar gel shift patterns in the column flow-through and initial washes (Figure 1, panels A and B, lanes 2 and 3). However, in contrast to the matrix only, three distinct RNA binding activities were eluted under conditions of increasing ionic strength from the dengue 3′SL RNA column. The 500 mM NaCl fraction (Figure 1B, lane 9) contained three ribonucleoprotein (RNP) bands (RNP1, 2 and 3), wherein the RNP2 complex was most predominant. The 1 M NaCl fraction contained mostly RNP1 (Figure 1B, lane 10). The RNP2 band in the 500 mM NaCl fraction and the RNP1 band in the 1 M NaCl fraction were highly reproducible.

**Figure 2:**
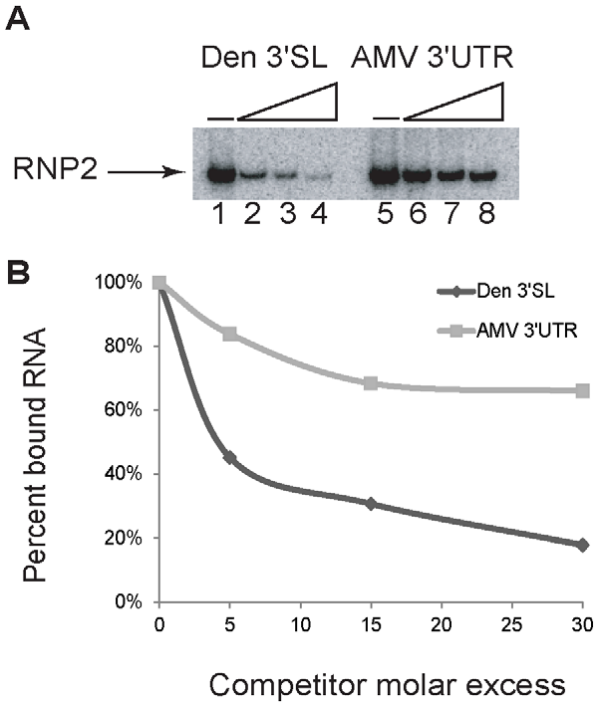
Binding specificity assessed by competition. (A) Electrophoretic mobility shift assay of a competitive RNA binding analysis. All reactions contained the same amount of radiolabeled dengue 3′ SL RNA and included the same volume of 500 mM affinity column chromatography eluate. Lanes 1 and 5 represent dengue 3′SL RNA plus protein extract, showing RNP2 without added competitor RNA. Lanes 2–4 represent competitor dengue 3′SL RNA added at 5, 15, and 30-fold molar excesses. Lanes 6–8 represent addition of competitor AMV 3′UTR RNA at a molar excesses of 5, 15 and 30 fold. (B) Quantification of labeled RNA in the bound fraction (RNP 2) from panel A normalized relative to the total amount of RNP2 in the absence of competitor (lanes 1 and 5, respectively). Upper trace: AMV 3′UTR RNA competitor; lower trace: dengue 3′ SL RNA competitor.

To extend the analysis, competitive binding assays were conducted to assess binding specificity. The dengue 3′ SL has significant secondary structure [4], [5], therefore the structured alfalfa mosaic virus (AMV) 3′ untranslated region (UTR) RNA [20] was used for comparative competition experiments. Radiolabeled dengue 3′SL RNA was added to the 500 mM affinity column eluates along with competitor RNA, represented by either unlabeled cognate dengue 3′SL RNA, or AMV 3′UTR RNA. The RNP2 band was detected in the absence of competitor RNA (Figure 2A, lanes 1 and 5). In the presence of a thirty-fold molar excess of dengue 3′SL competitor RNA, the amount of labeled RNA in the RNP2 complex was reduced by more than 80% (Figure 2A, lane 4; Figure 2B, lower trace), demonstrating effective competition. By comparison, a thirty-fold molar excess of the AMV 3′UTR was much less effective, reducing the amount of shifted RNP2 by only about 30% (Figure 2A, lane 8; Figure 2B, upper trace). The four-fold binding differential is evidence that proteins in the 500 mM eluate form specific complexes with the dengue 3′ SL RNA. Taken together, the data presented in Figures 1 and 2 demonstrate that dengue 3′ SL RNA interacting proteins were enriched by RNA affinity chromatography.

### Characterization of dengue 3′SL binding activities

**Figure 3:**
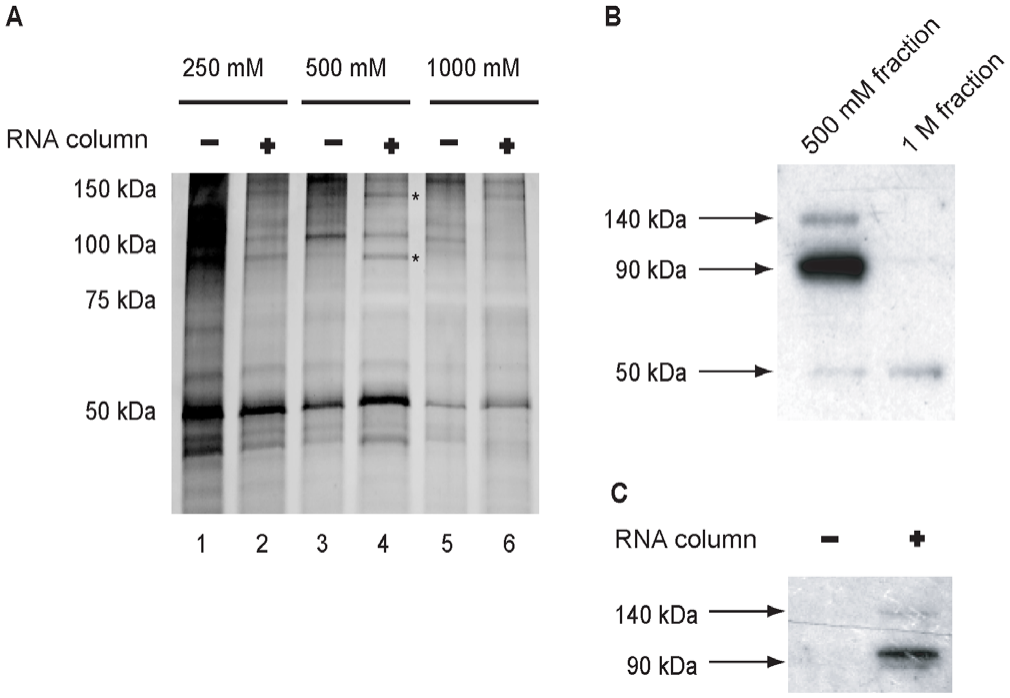
Protein composition and RNA binding activity of eluted fractions. Proteins in the column fractions were analyzed by SDS-PAGE followed by silver stain. (A) Lanes 1, 3 and 5 represent eluted fractions from the matrix-only column (-), while lanes 2, 4 and 6 represent fractions eluted from the RNA affinity column (+). Two stained bands were differentially present in the 500 mM sample eluted from the RNA column (lane 4, asterisks), and excised for MALDI-TOF MS analysis. (B) The 500 mM and 1 M column eluates were analyzed by northwestern blotting, using the dengue 3′ SL RNA as a probe. Left lane: 500 mM eluate; right lane: 1 M eluate. (C) Northwestern blot comparison of the 500 mM eluates from the control (-) and RNA-coupled (+) affinity columns. Left lane: 500 mM fraction from the control Sepharose column. Right lane: 500 mM fraction from the dengue 3′SL RNA column.

SDS-PAGE and silver staining, coupled with northwestern blotting, were used to characterize proteins eluted in the 500 mM RNA affinity chromatography eluate. By comparing the stain patterns from the control (no RNA) and dengue 3′ SL RNA columns, we observed that two distinct protein bands with approximate molecular weights of 140 kDa and 90 kDa were enriched in the dengue 3′SL RNA affinity column eluate (Figure 3A, compare lanes 3 and 4; asterisks). To determine if the stained 140 kDa and 90 kDa bands correlated with direct dengue 3′ SL RNA binding potential, a northwestern blot assay, using a radiolabeled dengue 3′ SL RNA probe, was performed. The data (Figure 3B) demonstrate a prominent signal in the 90 kDa region of the gel, along with lower intensity signals at 140 kDa and 50 kDa. Relatively weak signal was observed in the 50 KDa region of the 1M eluate separation (Figure 3B, right lane). As a specificity control, we compared 500 mM NaCl eluates from the control column (lacking bound RNA) and the dengue 3′ SL affinity column in the northwestern blot assay. The results demonstrate that the probe bound to the 140 kDa and 90 kDa bands in the RNA affinity column eluate (Figure 3C, right lane 2); however, no signal was present in the control column eluate (Figure 3C, left lane). The 140 kDa and 90 kDa bands seen in lane 1 of the northwestern analysis (Figure 3B) correlate with two bands of similar molecular weight observed in the silver stained gel (Figure 3A, lane 4), suggesting that proteins of these molecular weights bind the dengue 3′ SL RNA directly. These data are evidence that the 140 kDa and 90 kDa proteins bind directly to the dengue virus 3′ SL RNA.

### Identification of the dengue 3′ SL RNA binding proteins

The 140 kDa and 90 kDa protein bands were reproducibly enriched in the 500 mM fractions from RNA affinity column analyses (Figure 3A, compare lanes 3 and 4). In other experiments, SDS-PAGE electrophoretic resolution was improved by running a longer gel, revealing an additional 42 kDa band (data not shown). We analyzed the 140 kDa, 90 kDA and 42 kDa bands from two independent purifications by matrix-assisted laser desorption instrument time of flight (MALDI-TOF). The data are summarized in Table 1. MS-Fit [21] and Mascot software [22] identified the predicted peptides for the 90 kDa protein from Figure 3A as the interleukin enhancer binding factor 3 (ILF3), also known as nuclear factor 90 (NF90), DSRBP76, and nuclear factor associated with double stranded RNA (NFAR-1), among other names. The 140 kDa band was identified as the DEAH box polypeptide 9 (DHX9), more commonly known as RNA helicase A (RHA). MALDI-TOF MS analysis of the 42 kDa protein identified it as the interleukin enhancer binding factor 2 (ILF2), also known as nuclear factor 45 (NF45). Taken together, the results presented here demonstrate that NF90, NF45, and RHA were captured by the dengue 3′ SL RNA affinity column; moreover, the northwestern blot data are consistent with the hypothesis that NF90 and RHA bind directly to the dengue 3′SL RNA.

**Table 1:** Summary of MALDI-TOF MS results from the excised protein bands.

Band	MOWSEScore^a^	MassesMatched^b^	Coverage^c^	MW (Da)	Protein
**90 kDa**	115	100%	22%	74560	NF90
**140 kDa**	84	84%	13%	140869	RHA
**42 kDa**	182	89%	38%	44669	NF45

a The MOWSE score reflects the probability that the submitted masses are not a random match; scores greater than 76 (*P*<0.05) are considered significant.

b Number of masses that were matched with predicted peptides with high probability.

c Coverage refers to the percentage of the protein that was spanned by the predicted peptides.

### Cytoplasmic NF90 localization in the cytoplasm of dengue infected cells

**Figure 4:**
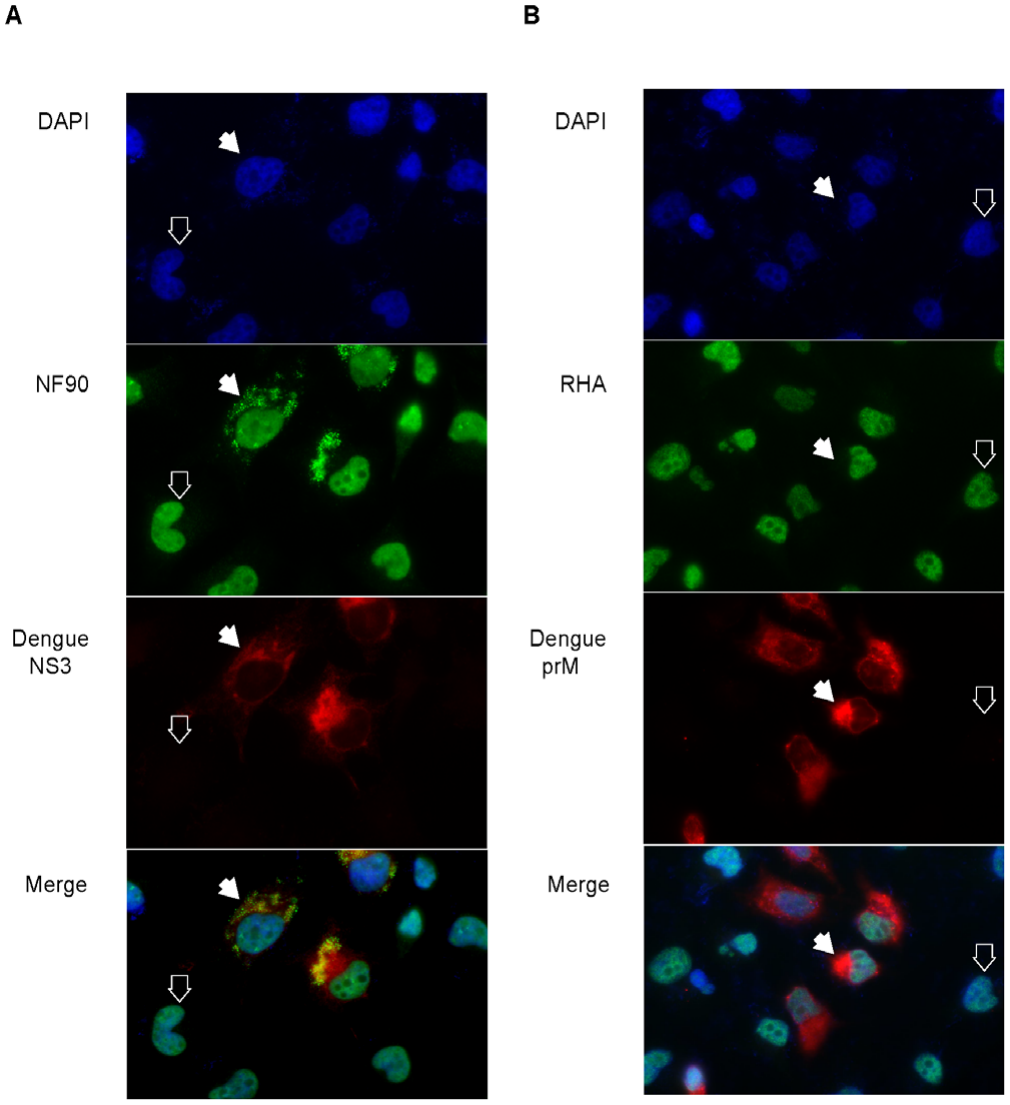
RHA and NF90 intracellular localization in uninfected and dengue virus-infected cells. Open arrows point to representative uninfected cells; filled arrows point to representative cells infected by dengue virus. (A) Cells were stained with the nuclear stain DAPI, with anti-NF90 mouse monoclonal antibody, and anti-dengue virus NS3 rabbit polyclonal antibodies. The merge panel shows an overlay of the DAPI, NF90 and NS3 signals. (B) Cells were stained with the nuclear stain DAPI, with anti-RHA rabbit polyclonal antibodies, and anti-dengue prM mouse monoclonal antibody. The merge panel shows an overlay of the DAPI, RHA and prM signals. Different dengue antibodies were used in panels A and B because of the requirement for secondary antibody specificities.

To begin to assess the potential functional significance of the identified proteins in the dengue virus life cycle, we examined NF90 and RHA cellular distribution in uninfected and dengue virus-infected HeLa cells using immunofluorescence imaging (Figure 4). DAPI nuclear staining identified all cells in the images (Figure 4, top panels) wherein the filled white arrows mark representative dengue-infected cells, and the open white arrows mark representative uninfected cells in all panels. The NF90 panel of Figure 4A depicts cells stained for NF90 (green color), and the dengue panel shows dengue NS3 protein (red color). We observed that detectable NF90 was exclusively nuclear in uninfected cells (open arrows), but both nuclear and punctate cytoplasmic staining was observed in dengue-infected cells (Figure 4A, NF90 panel, filled arrow). The merge panel suggests that in dengue infected cells, NF90 may colocalize to replication centers where NS3 is present. In the RHA panel of Figure 4B, we stained cells for RHA (green), and in the dengue panel for viral prM protein (red). In contrast to NF90, RHA staining was exclusively nuclear; with no differences observed when comparing infected (closed arrows) and uninfected (open arrows) cells (Figure 4B, RHA panel and prM panel). The merge panel confirmed the lack of detectable RHA relocalization of RHA to the cytoplasm of dengue-infected cells. These data strongly indicate that accumulation and/or relocalization of NF90 from the nucleus to the cytoplasm correlates with dengue virus replication. NF90's cytoplasmic localization in dengue virus-infected cells suggested a possible functional correlation with the viral life cycle; therefore, we focused further experiments on NF90.

### Supershift assays confirm the presence of NF90 in RNP2

**Figure 5:**
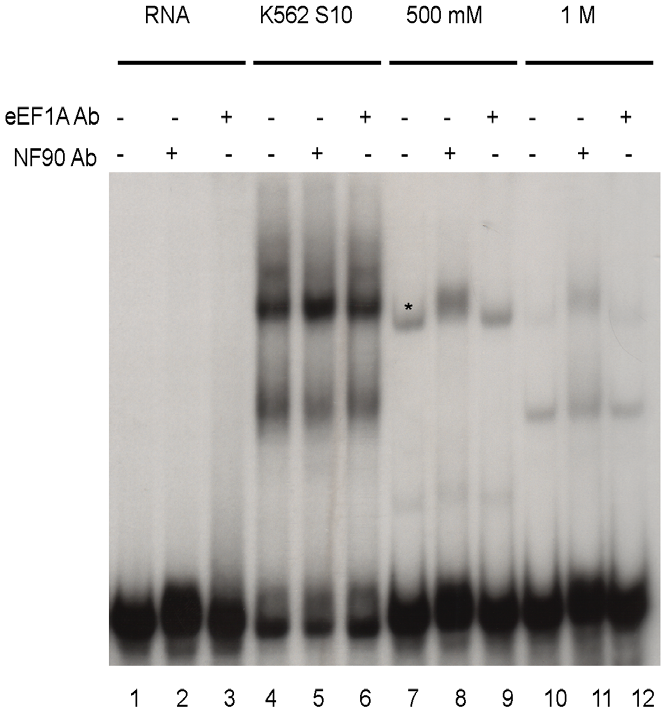
Supershift assay confirms the presence of NF90 bound to dengue 3′ SL RNA. A bandshift assay, as described for Figure 1, was performed after incubating radiolabeled RNA and proteins extracts in the absence of added antibody (lanes 1, 4, 7, 10), in the presence of anti-NF90 antibody (lanes 2, 5, 8, 11), or in the presence of anti-eEF1A antibody (lanes 3, 6, 9, 12). All reactions included the same amount of radiolabeled dengue 3′ SL RNA. Lanes 1–3: RNA only incubated in the absence (lane 1) or presence (lanes 2, 3) of indicated antibody. Lanes 4–6 represent bandshift assays using S10 extract from K562 cells in the absence/presence of the indicated antibody. Lanes 7–9 represent bandshift assays using the 500 mM fraction as protein source, in the absence/presence of the indicated antibody. An asterisk (lane 7) has been placed immediately above the RNP2 band previously identified in Figure 1B. Lanes 10–12 represent bandshifts assay using the 1 M fraction as a protein source, in the absence/presence of the indicated antibody.

A supershift EMSA identifies a protein in an RNP complex by specific antibody binding, decreasing the electrophoretic mobility of the resulting antibody-RNP complex as compared to the identical RNP complex lacking bound antibody. We performed supershift experiments to assess the presence of NF90 and eEF1A proteins in the RNP complexes. eEF1A was analyzed because of a previous report that it binds the West Nile virus and dengue virus 3′ SL RNAs, with possible roles in West Nile virus negative strand RNA synthesis [23], [24]. Control immunoblot experiments confirmed that anti-NF90 and anti-eEF1A antibodies recognized their target proteins (data not shown); moreover, neither of the antibodies affected the migration of the dengue 3′ SL RNA alone (Figure 5, lanes 2 and 3). The data shown in Figure 5, lanes 7 and 10, reproduce the shift pattern observed in Figure 1B, lanes 9 and 10. The asterisk in Figure 5, lane 7 is placed above RNP2. When the NF90 polyclonal antibody was added, we observed an RNP2 supershift in the 500 mM fraction sample (Figure 5, lane 8), but not in the more rapidly migrating RNP1 band (Figure 5, compare lanes 10 and 11). Supershifts were not observed when the eEF1A polyclonal antibody was used with either the 500 mM fraction (Figure 5, lane 9) or the 1 M fraction (Figure 5, lane 12). eEF1A protein was readily detected by western blotting using unfractionated cell extracts; however, it was not detected in the 1 M eluted proteins (data not shown) or by supershift (Figure 5, lanes 9 and 12), suggesting that the 50 kDa band observed in Figure 3A does not include detectable eEF1A. The shift patterns observed using unfractionated K562 cell S10 extracts were not altered by the addition of either antibody (Figure 3; compare lane 4 with lanes 5 and 6), suggesting that the shifted bands (lanes 4–6) represent non-specific RNA-protein complexes that obscured specific supershifts (lane 8), or that specific shifts/supershifts could not be detected without the concentration effect provided by affinity chromatography. Together, the data presented in Figures 1, 2, 3, 5 and Table 1 suggest that NF90 is a dengue RNA binding protein found in the EMSA RNP2 complex (Figure 1B). The accompanying NF90 relocalization observed in dengue virus-infected cells (Figure 4) further led us to hypothesize that NF90 may have a functional role(s) in the dengue life cycle.

### NF90 depletion decreases intracellular dengue virus RNA and protein levels

**Figure 6:**
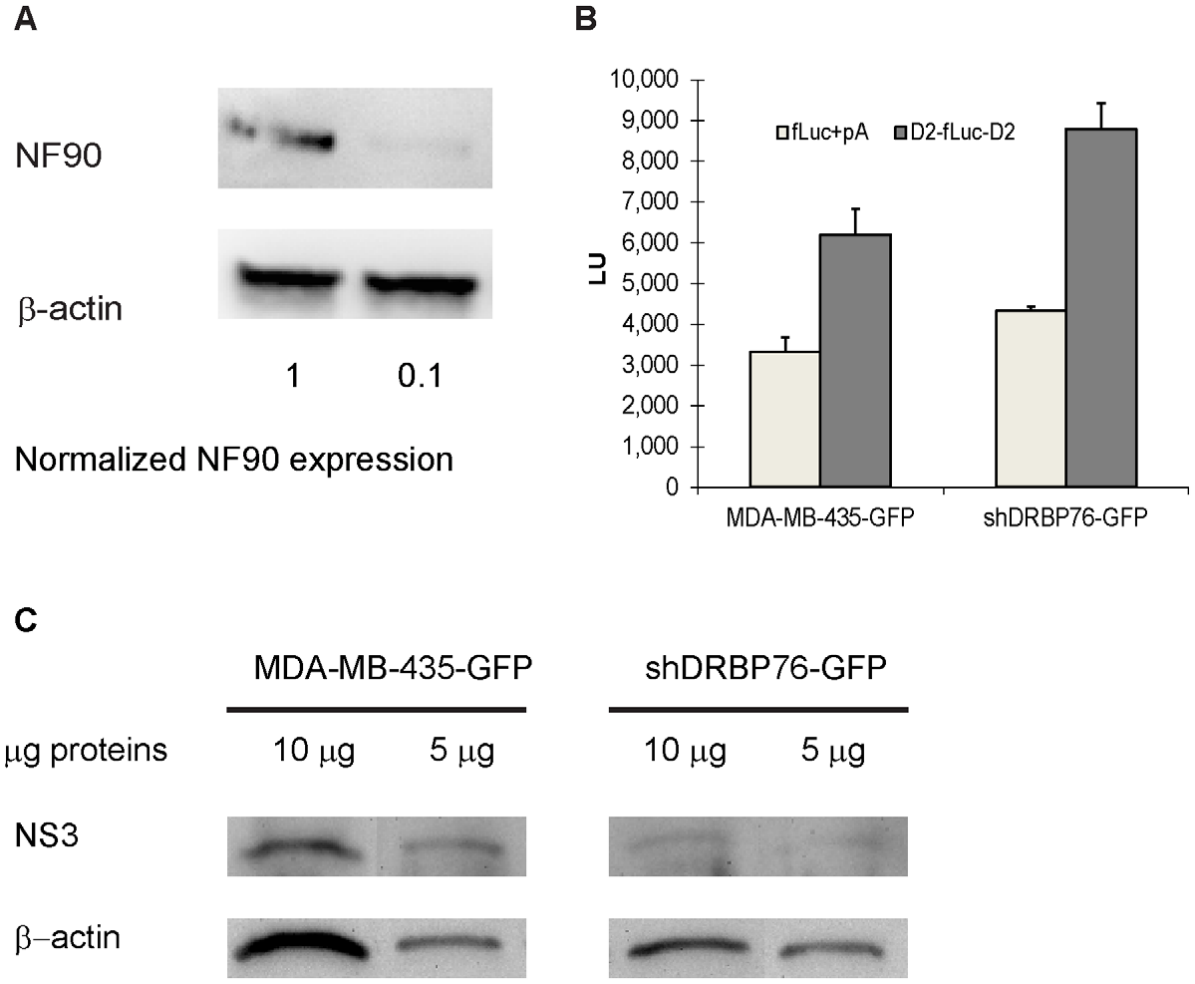
NF90 depletion decreases intracellular levels of dengue virus proteins. (A) Representative western blot showing NF90 levels in control MDA-MB-435 cells and in NF90-depleted shDRBP76-GFP cells. Extracts were prepared from the cells, analyzed by SDS-PAGE, transferred to nitrocellulose membranes, and then probed concurrently with anti-NF90, and anti-β-actin antibodies. (B) Translational capacity of NF90 depleted cells. Wild type (MDA-MB-435-GFP) and NF90 shRNA knockdown cells (shDRBP76-GFP) cells were transfected with reporter mRNAs. The figure shows reporter luciferase units (LU) expressed from the reporter mRNAs in the two cell types. Fluc + pA: firefly luciferase reporter mRNA with cognate 5′ and 3′ UTR sequences and a 3′ polyA tail; D2-fluc-D2: firefly luciferase coding region flanked by dengue virus serotype 2 5′ and 3′ UTR sequences (C) Viral NS3 protein levels detected by western blotting 48 hrs after dengue virus infection of wild type and NF90 knockdown cells. NS3 and actin proteins were detected by western blotting using 10 µg and 5 µg loading amounts of total cell lysate.

Vumbaca *et al.* (2008) described the preparation of NF90-depleted cells by stable shRNA expression to generate knockdown cells (shDRBP76-GFP) from the parent MDA-MB-435-GFP cells (48). Western blot analysis showed that NF90 levels in the knockdown cells were reduced to approximately 10% of those observed in control cells (Figure 6A, compare NF90 bands in lanes 1 and 2, relative to the actin protein loading controls). We performed luciferase reporter translation assays to confirm that the translational capacity of the NF90 knockdown cells was not diminished. Messenger RNAs containing the luciferase coding region flanked by the cognate UTRs or the dengue UTRs were transcribed *in vitro* and transfected into the wild type and NF90 knockdown cells, followed by assay for luciferase activity. The results suggest that accumulation of reporter luciferase proteins was statistically indistinguishable when comparing the wild type and NF90 knockdown cells (Figure 6B). These results confirm the observation by Vumbaca *et al.* that NF90 depletion does not affect overall translational capacity in these cells as revealed by polysome analysis [25]. The data (Figure 6A and 6B) justify the use of the NF90-depleted cells to evaluate NF90's role in regulating dengue RNA accumulation and production of infectious virus.

MDA-MB-435-GFP and shDRBP76-GFP NF90 knockdown cells were infected with type 2 dengue virus at a multiplicity of infection (MOI) of 0.5. Total protein isolated at 48 hours post-infection was analyzed by Western blot to examine the presence of dengue virus NS3 protein (Figure 6C). Visual inspection of the data suggested that viral NS3 levels were diminished in the NF90-depleted cells; however, accurate quantification was hindered by the small amounts of NF90 protein detected in the extracts from shRNA knockdown cells (Figure 6C, right panel). To generate quantitative data, we used real-time RT-PCR to assess the accumulation of dengue virus RNA in dengue virus-infected wild type MDA-MB-435-GFP and shDRBP76-GFP NF90 knockdown cells. Table 2 shows the raw average and standard deviation of *C_T_* values of triplicate wells from three independent experiments, assayed in triplicate. Processing these data using the ΔΔ*C_T_* method yields the normalized dengue expression ratios presented in Table 2. At each successive 12 hour time point post-infection, the relative dengue RNA levels in the shDRBP76-GFP NF90 knockdown cells were 0.42, 0.52, 0.28 and 0.40 as compared to those in the MDA-MB-435-GFP cells. Statistical significance was determined using the randomization method described by Pfaffl *et al*. [26], and the resulting *P*-values are shown in Table 2. These data are consistent with the visual inspection of the NS3 protein accumulation results (Figure 6) and suggest that virus replication and/or viral RNA translation are reduced by 50–70% when NF90 protein is depleted.

**Table 2:** Quantification of dengue virus RNA levels and normalized RNA expression ratios in dengue virus-infected cells.

	MDA-MB-435-GFP control		shDRBP76-GFP knockdown		Normalized dengue	
h.p.i.	Dengue *C_T_*	β-actin *C_T_*	Dengue *C_T_*	β-actin *C_T_*	RNA expression ratio^a^	*P*-value
12	29.9±1.0	21.7±0.5	30.3±0.8	20.9±0.7	0.42	<0.001
24	26.5±1.2	21.3±0.8	28.4±1.0	22.1±0.6	0.52	<0.02
36	25.0±0.6	22.4±0.6	26.7±0.9	22.2±0.6	0.28	<0.001
48	26.4±0.3	22.8±1.0	27.2±0.6	22.3±0.8	0.40	<0.001

C_T_ values represent the mean ± standard deviation (SD) of three independent experiments, performed in triplicate. C_T_ values were analyzed using the Relative Expression Software Tool (REST), wherein expression ratios are generated and tested for significance by a randomization test [26].

a Normalized dengue RNA expression represents the ratio of dengue RNA levels in NF90 knockdown cells relative to dengue RNA levels in control cells, each normalized to actin RNA levels.

### NF90 depletion decreases production of functional dengue virus

**Figure 7:**
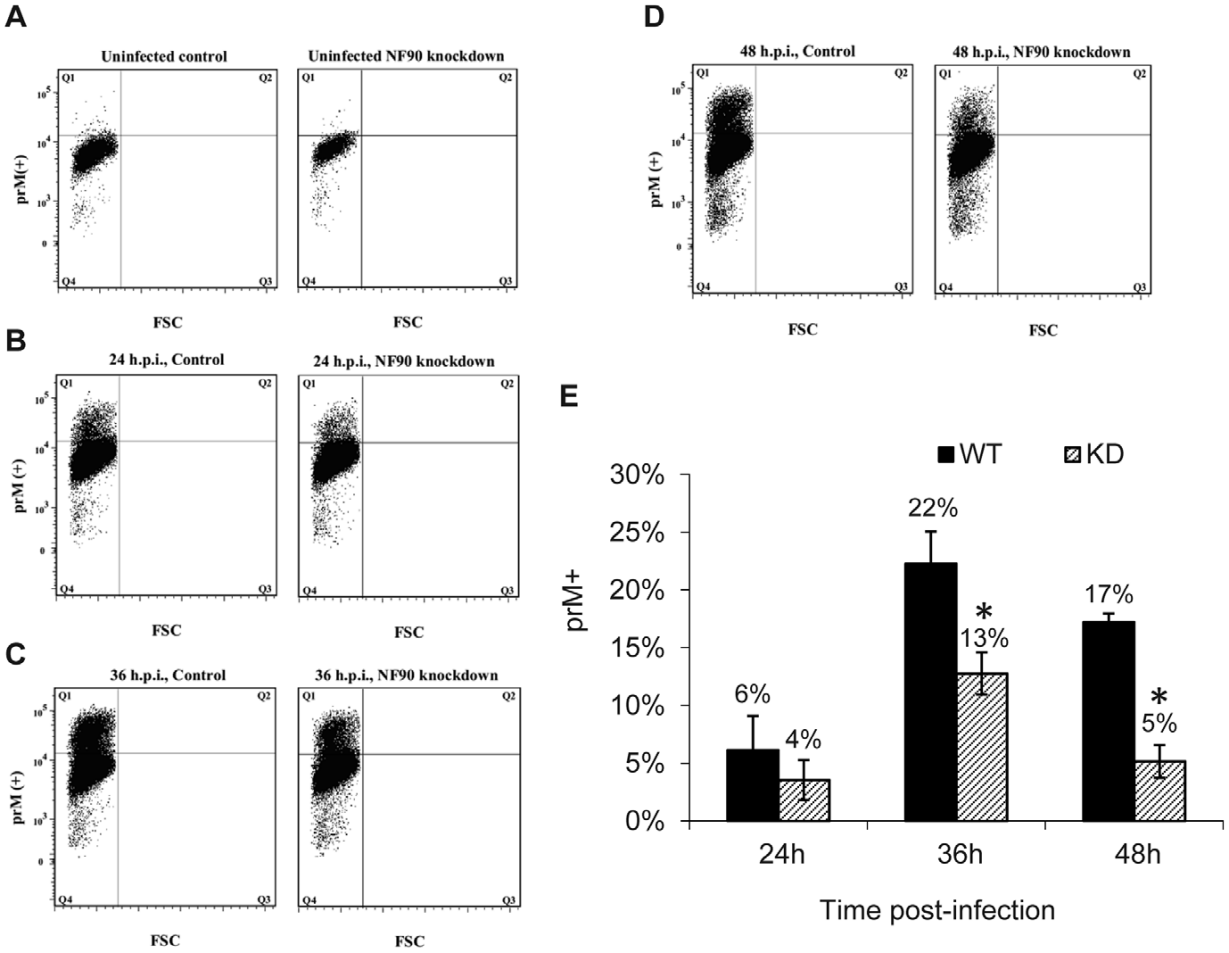
NF90 depletion reduces production of infectious dengue virus. Supernatants from WT and NF90-depleted cells infected with dengue virus (multiplicity of infection: 0.5) were used to infect DC-SIGN-expressing K562 cells (12, 46). At 14 hours post-infection, cells were harvested and analyzed for viral infection by flow cytometry using antibodies against dengue virus prM. Panels A-D are representative dot plots from the assay of a single set of supernatants from WT (MDA-MB-435-GFP) and KD (shDRBP76-GFP cells), FSC: forward scattering. (E) Bar graph summarizing the results of three independent experiments. Asterisks represent statistical significance at *P*<0.02.

To extend the analysis, we used a flow cytometry-based assay to quantify infectious dengue particles released by MDA-MB-435-GFP and shDRBP76-GFP cells at 24, 36, and 48 hours after infection with dengue virus. Supernatants from infected cells were used to infect K562 cells that express the lectin DC-SIGN, which is important for dengue virus adherence and entry [27]. After a 14-hour incubation period, flow cytometry was used to score the number of infected K562 cells, identified by positive staining for dengue prM protein [28] (Figure 7). Figure 7A serves as a representative negative control and demonstrates the sensitivity limit. The amounts of virus in the supernatants were not significantly different in MDA-MB-435-GFP vs. shDRBP76-GFP cells at 24 hours post infection in three independent experiments, when only about 5–7% of cells were infected (Figures 7B and 7E). The quantified differences between infectious particles produced by dengue-infected control and NF90 knockdown cells was statistically significant at the 36 and 48-hour time points post-infection, with the NF90-depleted cells lagging the control cells by approximately 39% and 70%, respectively. Taken together, the data presented in Figures 6 and 7 suggest that NF90 has a positive functional role in the life cycle of dengue virus.

## Discussion

Flavivirus 3′ untranslated RNA regions are known to have important regulatory significance for viral RNA replication and translation [29]; moreover, the predicted secondary structures of their 3′ terminal stem loops (3′ SL) are conserved [5]. We hypothesized that these conserved regions may interact with host proteins that regulate viral RNA translation and/or replication, and here we describe the biochemical identification of several host proteins that co-isolate with the dengue virus 3′ SL RNA: RHA (DHX9, NF90 (NFAR-1/DRBP76), and NF45 (ILF2). The RNA column fractions containing NF90 and RHA yielded positive RNA binding signals by northwestern blot with the dengue 3′SL RNA probe (Figure 3B), consistent with their identification as RNA binding proteins containing double strand RNA binding motifs [30]. NF90 also has an RGG single strand RNA binding domain; however, its functional significance has not been demonstrated [30]. NF45 does not have an RNA binding domain; rather, it forms a heterodimer with NF90 [31].

In related work, NF90 and RHA have been found in complex with the adenovirus associated RNA II (VA _II_) [32] and with the 5′ and 3′UTR regions of both the pestivirus BVDV RNA [16] and the hepacivirus HCV [17] RNAs, suggesting that these proteins could have conserved functions for the *Flaviviridae*. A novel feature of our work is that NF90, RHA and NF45 proteins are shown to be highly enriched by affinity chromatography using a flavivirus 3′ stem-loop RNA. Although dengue, BVDV, and HCV are all members of the viral family *Flaviviridae*, viral RNAs from the flavivirus genus (dengue, West Nile) are distinct from BVDV and HCV because they have a 5′ m7GpppG cap, and also require complementary 5′ and 3′ nucleotides sequences (cyclization sequences) for their replication [6], [33], [34]. While all members of the *Flaviviridae* have highly structured RNAs, the predicted secondary structures of their 3′ terminal regions are distinct [5], [35], [36]. Published data describe other proteins found to bind the dengue virus 3′ SL region. Paranjape and Harris reported that Y box-binding protein 1 binds the dengue virus 3′SL and mediates antiviral effects [37]. In addition, UV-crosslinking experiments suggested that purified La protein and polypyrimidine tract binding protein (PTB) interact with the dengue virus 3′SL [38].

Our analysis focused on highly represented proteins that eluted from the dengue 3′ SL affinity column and were identified by SDS-PAGE staining (Figure 3A, lane 4), followed by northwestern blot experiments (Figures 3B and 3C). Additional dengue 3′ SL binding proteins may have been present in the affinity column eluates that were not readily distinguishable in the stain patterns or northwestern blots. The RNA affinity column and EMSA data revealed the RNP2 complex observed in Figure 1B. Competitive binding experiments (Figure 2) demonstrated four-fold differential relative binding specificity for dengue 3′-SL binding proteins in the RNP2 complex, and 90 kDa/140 kDa proteins were apparent as RNA binding proteins in the northwestern analysis (Figure 3B and 3C), correlating with the highly represented NF90 and RHA peptides identified by mass spectrometry (Table 1). We confirmed the presence of NF90 in the RNP2 complex by supershift analysis (Figure 5); however, we did not observe comparable supershifts with the anti-RHA antibody, providing the initial justification for focusing functional analyses on NF90. A further justification for narrowing our functional analyses to NF90 is that NF90 alone showed a partial relocalization to the cytoplasm of dengue virus-infected cells (Figure 4), and we hypothesized that the relocalization may have regulatory significance for dengue virus RNA translation or replication.

NF90 relocalization has also been observed in BVDV-infected cells, in HCV replicon-transfected cells (24), and in cells undergoing a stress response [25]. NF90 shuttles between the cytoplasm and nucleus of activated T-cells [39]. As insight into the localization mechanism, Parrott and Mathews reported that cytoplasmic NF90 is phosphorylated, and that the phosphorylation disrupts NF90's interactions with nuclear proteins, thereby releasing it into the cytoplasm [40]. Although we did not observe cytoplasmic RHA by immunofluorescence (Figure 4), RHA has been shown to shuttle between the nucleus and the cytoplasm in HIV infections [41] and in foot and mouth disease virus infections [42]. In addition, Isken *et al* ***.*** reported that cytoplasmic RHA was detected after HCV replicon transfection [17]. Although not detected by immunofluorescence, cytoplasmic RHA was detected by mass spectrometry after enrichment by affinity chromatography methods that concentrate bound ligands. The cytoplasmic localization of NF90 in dengue-infected cells was clear by immunofluorescence, while undetectable for RHA (Figure 4). However, we cannot completely rule out the possibility that low levels of relocalized cytoplasmic RHA, below detection levels by immunofluorescence, may have affected the dengue life cycle. Alternatively, the differential localization of RHA in dengue and HCV infections might suggest distinctive roles for RHA in the respective viral life cycles.

To test the hypothesis that NF90 has regulatory roles in dengue virus RNA translation and/or replication, we used shRNA-mediated protein depletion to examine viral RNA and protein (NS3) expression, as well as release of infectious dengue virus particles. Planning for the NF90 knockdown experiments was tempered by prior reports of technical challenges, including cell death caused by NF90 RNA interference [17], and embryonic lethal [43] or perinatal lethal [44] mice from NF90 gene disruption experiments. Alternatively, other laboratories reported success in siRNA depletion of 50%–95% of detectable NF90 without significant cell death [43]. The range of responses to NF90 depletion may, therefore, reflect the behaviors of different cell types.

Here, we have successfully used human melanoma cells (MDA-MB-435) that express NF90 shRNA constitutively to significantly reduce NF90 levels [25]. Under these conditions, viral RNA, viral NS3 protein, and released infectious virus, were correspondingly diminished in the dengue-infected and NF90-depleted cells, as compared to melanoma cells lacking the shRNA. Taken together, the biochemical binding data and the functional data strongly suggest that NF90 is a specific dengue 3′ SL binding protein that is a positive regulator of the dengue virus life cycle. Isken *et al.* concluded similarly that NF90 is a positive regulator of BVDV and HCV replication [16], [17]. Conversely, Barber *et al.* reported that cells depleted of NF90 show enhanced susceptibility to vesicular stomatitis virus (VSV) and influenza virus infection [43], suggesting that NF90 negatively regulates VSV and influenza infections. The mechanisms underlying these seemingly opposite roles for NF90 have not been defined; however, VSV and influenza viruses are negative strand polarity viruses and replicate in the cell nucleus, while viruses in the *Flaviviridae* are positive-stranded and replicate in the cytoplasm. NF90 could have distinct functions in individual cellular compartments, leading to differential effects on virus replication.

Accumulated evidence from the literature provides guidance for future work to define the mechanism of NF90′s positive regulatory role in dengue virus replication. NF90′s functions have been coupled to those of NF45, suggested by experiments demonstrating that their expression is regulated coordinately [31]. NF90 has been linked to nucleo-cytoplasmic RNA export [43] and also to selective RNA localization, as reported for Tau mRNA localization in neurons [45]. By analogy, NF90 might have a role in localizing dengue virus RNA to replication centers. The NF90/NF45 complex has been linked to negative regulation of micro RNA (miRNA) processing [46]. The corollary with our data would predict the identification of a miRNA that is itself a negative regulator of dengue RNA translation or replication. In the absence of knockdown, NF90/NF45 would repress the expression of this negative regulator, permitting virus production. However, when NF90/NF45 is coordinately depleted, then the negative regulator miRNA would be processed and available to down-regulate dengue RNA translation. The NF90/45 complex has also been reported to inhibit translation initiation at the internal ribosome entry site (IRES) of rhinovirus [47].

NF90's role in RNA stabilization has been described in several recent publications, suggesting that NF90 could have a role in increasing the dengue RNA half-life. NF90 binding has been reported to enhance the stability of IL-2 mRNA [46], p21WAF1/CIP1, MyoD mRNAs [44], and VEGF mRNA [25]. Although our data demonstrate that NF90 binds the dengue 3′ SL RNA, dengue RNAs do not have obvious AU-rich instability elements (AREs) that have been described for the IL-2 mRNA [46], and the VEGF mRNA [48]. The absence of available NF90 to bind the dengue 3′SL during an shRNA-mediated NF90 depletion experiment could correlate with a faster turnover of the dengue mRNA and account for decreased viral RNA and NS3 levels observed in the infected shDRBP76-GFP NF90 knockdown cells. The potential role of NF90 as a dengue RNA stabilizer remains to be explored. An alternate or perhaps complementary NF90 function would be to recruit dengue mRNA into polysomes for efficient translation, as reported for VEGF [25], or to coordinate viral RNA translation and replication [16]. Our reporter assay results, however, suggest that NF90 depletion does not affect the translation of a reporter containing the dengue UTRs. It is possible that additional motifs in the genomic viral RNA (not present in the reporter construct) are involved in NF90's potential function in translation.

A limitation of RNA interference approaches for studying proteins with multiple functions, such as NF90, is that conclusions about specific regulatory mechanism(s) controlling virus replication can only be inferred. Ablating the NF90 protein binding site by mutating the dengue 3′ SL RNA could yield greater precision in linking replication with formation of a viral RNA-protein complex [16]; however, 5′ and 3′ viral RNA domains have roles in both replication and translation [13], [49], and mutagenesis in these regions requires a very cautious approach to avoid introducing artifacts. The data reported here strongly suggest that NF90 depletion diminishes the production of infectious dengue virus by more than half, which is significant for a pathogen that places more than a billion people at risk worldwide. Direct NF90 depletion is likely not a viable therapeutic approach because of its multifunctional properties; however, further analysis may identify a specific site(s) in NF90-mediated regulatory pathways that show greater specificity for interrupting the dengue virus life cycle.

## Materials and Methods

### Template cloning and plasmid descriptions

The terminal 3′ end stem loop (3′SL) of dengue virus was amplified by DNA thermal cycling from the clone 2A, which contains the 5′ and 3′ untranslated regions (UTRs) from dengue virus type 4 strain 814669 (gift from Dr. C. J. Lai, NIH) [50]. The *in vitro* transcription vector used was pHST70, which is similar to the pHST0 vector described previously [51], except that the bacteriophage SP6 promoter was replaced by the bacteriophage T7 promoter sequence. The PCR fragment was ligated into plasmid pHST70, previously digested with *HindIII* and *XmaI*, creating plasmid pDen3′SL. Plasmid pHST442, encoding the full length alfalfa mosaic virus (AMV) 3′UTR, has been described previously [20].

### RNA transcriptions

For RNA transcriptions, plasmids pDen3′SL and pHST442 were linearized with *SmaI*. The T7 Megashortscript kit (Ambion) was used to transcribe the RNAs. For transcription of radiolabeled RNA, reactions were supplemented with 20 µCi [α-^32^P]-UTP (Perkin-Elmer). Dengue 3′SL RNA was purified by denaturing gel electrophoresis. The RNA was visualized by ultraviolet light shadowing, followed by excision of the gel slice and RNA elution overnight at 4°C in crush and soak buffer (500 mM NH_4_OAc, 10 mM MgOAc_2_, 1 mM EDTA, 0.1% SDS) [52]. The supernatant was extracted once with phenol:chloroform (1∶1), followed by precipitation of the nucleic acids with ethanol. The concentration and specific activity of radiolabeled RNAs were determined by absorbance spectrometry and liquid scintillation counting, respectively.

### Cells and viruses

K562 cells and HeLa cells were purchased from the American Type Culture Collection (ATCC) (Manassas, VA) and maintained in culture media (DMEM, 10% FCS and antibiotic/antimycotic (Invitrogen) at 37°C in 5% CO2. C6/36 (from ATCC) were maintained in RPMI 1640, 10% FCS and antibiotic/antimycotic (Invitrogen) at 28°C in 5% CO2. MDA-MB-435-GFP cells, generated by transfecting parental cells with pcDNA3.1/Neo (Invitrogen) vector [53], and cells expressing NF90-specific shRNA (shDRBP76-GFP) [25] were a gift from Dr. Kevin P. Claffey (University of Connecticut Health Center). The cells were kept in DMEM, 10% FCS and antibiotic/antimycotic (Invitrogen) at 37°C in 5% CO2. Stable transfectants were selected through growth in culture medium containing 1 mg/mL Geneticin (Invitrogen) and confirmed by GFP fluorescence.

Dengue virus type 2, strain New Guinea C, (gift from Dr. Irene Bosch) was amplified by infecting C6/36 mosquito cells in RPMI infection media (RPMI 1640, 2% FCS and antibiotic/antimycotic), followed by incubation at 28°C for 4 days or until cellular cytopathic effects became apparent. The virus-containing supernatant was harvested, followed by removal of cellular debris by brief centrifugation, and stored as aliquots at -80°C.

### RNA affinity chromatography

To purify dengue 3′SL binding proteins from the K562 cell S10 extracts, affinity chromatography using coupled dengue 3′SL RNA was performed. To prepare S10 lysates, K562 cells were harvested from confluent cultures grown in T150 flasks after gentle pipetting to resuspend the cells evenly. Cells were washed once in PBS and resuspended in 1X packed cell volume of hypotonic lysis buffer (20 mM Tris pH 7.5, 10 mM KCl, 1.5 mM MgOAc_2_, 7 mM β-mercaptoethanol, and a cocktail of protease inhibitors (Invitrogen)). After incubation on ice for 10 min, cells were homogenized using a Dounce homogenizer. The lysate was cleared by centrifugation at 10,000×*g* for 10 min at 4°C. The KCl concentration in the supernatant was adjusted to 100 mM. The S10 extract was then complemented with 200 µg/mL of an RNA poly-C homopolymer (GE Lifesciences) to adsorb nonspecific binding proteins and reduce background binding to the RNA affinity column.

Approximately 300 µg (∼1 mL column volume) of CNBr activated Sepharose 4B (GE Lifesciences) was swelled in 10 mL of 1 mM HCl for 1 hr at room temperature. The swelled matrix was then washed with 100 mL of ice-cold 1 mM HCl using a Buchner filter with a fritted disc. The matrix was resuspended from the Buchner filter using 10 mL of 10 mM Tris pH 6.8. One hundred micrograms of *in vitro-*transcribed dengue 3′SL RNA was immediately added to the slurry and covalently coupled by rocking at 4°C overnight. As a control for the specificity of the purification, an equal amount of Sepharose 4B without RNA was prepared similarly to the Sepharose-RNA matrix. The Sepharose-RNA and Sepharose-only beads were then equilibrated in buffer A (10 mM Na_2_HPO_4_ pH 7.2, 100 mM NaCl, 0.1 mM EDTA, 5% glycerol and 7 mM β-ME) by rocking at 4°C overnight. After equilibration, beads were packed into a Bio-Rad Poly-Prep column (0.8 cm×4 cm, 2 mL bed volume).

Approximately 8 mL of K562 S10 cell extract (prepared as described above) was passed through the Sepharose-only column four times, sequentially, to pre-clear the extract. The pre-cleared extract was then applied to the Sepharose-RNA column four times. The column was then washed with 10 column volumes of buffer A to remove weakly bound proteins and proteins retained in the matrix. Proteins were step-eluted using 250 mM, 500 mM, 1M and 2M NaCl in 15 mM Tris pH 7.5. Fractions were desalted and concentrated using Nanosep 3K filters (Pall), and the proteins were diluted in buffer A to 100 µL. Fractions were tested for dengue 3′SL RNA binding in an EMSA assay as described above. To analyze the composition of the purified fractions, proteins were separated by SDS-PAGE in 10% pre-cast BioRad gels. Silver staining was done using the BioRad Silver Stain kit, according to the manufacturer's recommendations.

### Electrophoretic mobility shift assay (EMSA)

EMSA was performed as previously described [20]. Dengue 3′SL RNA, radiolabeled with [α-32P]-UTP, was diluted in renaturation buffer (10 mM Tris pH 7.5, 50 mM NaCl, 3 mM MgCl2 and 0.1 mM EDTA) followed by heating at 90°C for 2 min and then quick cooling on ice to disrupt aggregates. Binding reactions were set at room temperature in binding buffer (10 mM Na2HPO4 pH 7.2, 60 mM KCL, 1 mM EDTA, 7 mM β-mercaptoethanol, 5% glycerol, and 50 ng/µL of poly-C) in a volume of 10 µL for 20 min. Reactions were analyzed by electrophoresis into a non-denaturing 10% polyacrylamide gel, which was dried and exposed to film overnight. For supershift assays, binding reactions were set as described above. After 15 min of incubation, antibodies to NF90 (gift from Dr. Michael Mathews, Univ. Med. Dent. New Jersey) or eEF-1A (gift from Dr. William Merrick, Case Western Reserve University) were added to the reaction (1 uL each). After a further 15 min of incubation, the complex was resolved by electrophoresis into a 10% non-denaturing polyacrylamide gel as described above.

The protocol for competitive RNA binding assays was similar to that of bandshift assays (EMSA), with the difference that varying amounts of non-radioactive competitor RNAs were mixed with the radiolabeled dengue 3′SL RNA prior to the addition of proteins. For quantification, gels were exposed to phosphorimager screens (GE Lifesciences) overnight. The screens were scanned using the Storm 8600 instrument (GE Lifesciences). The results were analyzed using ImageQuant software (GE Lifesciences).

### Northwestern analysis

Northwestern analysis was performed as described [54] with minor modifications. Proteins were separated by SDS-PAGE (10% pre-cast gels [BioRad]), followed by electrophoretic protein transfer to nitrocellulose membranes overnight at 4°C and 30 V. Non-specific interacting sites on the membranes were blocked by incubating in 5% Blotto (Pierce) in PBST (PBS +0.1% Tween-20) for 1 hr at room temperature. The membranes were then washed in HBB buffer (25 mM HEPES-KOH pH 7.5, 25 mM NaCl, 5 mM MgCl2 and 7 mM β-ME) for 10 min. Proteins were denatured and renatured on the membrane by two successive washes in HBB buffer containing 6 M guanidine chloride, followed by washing once each in 3 M, 1.5 M, 0.75 M, 0.375 M and 0.187 M guanidine chloride in HBB for 10 min. Membranes were then washed in HBB, followed by 2 washes in HYB100 (20 mM HEPES-KOH pH 7.5, 200 mM KCl, 2.5 mM MgCl2, 100 µM EDTA, 0.05% NP40 and 7 mM β-ME). Approximately 1×10 6 CPM of renatured *in vitro* transcribed 3′SL dengue RNA in HYB100 were used to probe the membranes for 4 hr at room temperature. Following this incubation, membranes were washed three times using HYB100 buffer for 10 min, wrapped in Saran wrap, and exposed to film overnight.

### Mass Spectrometry Analysis

To identify proteins retained specifically on the dengue 3′SL RNA Sepharose column, SDS-PAGE gel fragments were excised, digested with trypsin and analyzed by matrix assisted laser desorption instrument time of flight (MALDI-TOF) mass spectrometry (MIT Center for Cancer Research Biopolymers Laboratory). Peptide fragments were analyzed using the MS-Fit program [21]. Mascot software was used for refinement and statistical analysis of the MALDI-TOF data [22].

### Immunofluorescence imaging analysis

HeLa cells were used to analyze protein localization because MDA-MB-435–GFP cells express green fluorescent protein, complicating immunofluorescence analysis. HeLa cells were seeded into 12-well tissue culture plates containing sterile glass cover slips and grown to 70%–80% confluence. Cells were then infected with type 2 dengue virus, New Guinea C strain, using infection media (DMEM, 2% FCS and antibiotic/antimycotic) at a multiplicity of infection (m.o.i) of approximately 0.5. Virus was then removed and replaced with 1 mL of culture medium, and the cells grown for 48 hr at 37°C. Cells were washed three times with cold PBS and fixed with 4% paraformaldehyde in PBS for 10 min at room temperature. After fixation, cells were washed 3 times with PBS and permeabilized/blocked with two incubations of 10 min in 1%BSA/0.1% Triton X-100/PBS (PBSAT).

To image NF90 and dengue in the same cells, a mouse monoclonal anti-DRBP76 antibody (BD Biosciences) was used with a rabbit anti-dengue NS3 protein polyclonal antibody (provided by Dr. R Padmanabhan, Georgetown University). Similarly, to image dengue and RHA, a rabbit anti-DHX9 polyclonal antibody (Abcam) was used with a mouse anti-dengue monoclonal antibody (U.S. Biologicals). The secondary antibodies were goat anti-rabbit IgG coupled to Alexa 488 (Molecular Probes) and goat anti-mouse IgG coupled to Alexa 594 (Molecular Probes). Cells were incubated with 500 µL of primary antibody (1∶200) in PBSAT for 45 min at room temperature, followed by three washes with PBS. Secondary antibody was diluted (1∶500) in PBSAT, added to the cover slips, and incubated for 45 min at room temperature. After three washes with PBS, cover slips were then removed and mounted on glass microscope slides with ProLong Gold/DAPI (Invitrogen). Images were captured using MetaMorph (Molecular Devices) software and data analysis was carried out with ImageJ (NIH).

### Reporter constructs and RNA transcription

The firefly luciferase cDNA was obtained from Promega. Using PCR, a 50-nucleotide poly(A) tail was cloned downstream of the luciferase coding region. The complete 5′ UTR with the first fifteen codons of the dengue virus capsid protein and complete 3′ UTR of the dengue virus RNA were amplified individually from the cDNA clone (D2NGC strain, gift of Dr. Barry Falgout, FDA) and cloned upstream and downstream, respectively of the firefly luciferase coding region, without the poly(A) tail. Reporter construct RNAs were transcribed from linearized DNA templates using the mMessage mMachine transcription kit (ABI). MDA-MB-435-GFP and shDRBP76-cells were each transfected with 100 ng of reporter RNA, and after three hours, luciferase activity was measured using the Luciferase Assay System (Promega). Samples were read in a TD20/20 luminometer (Turner Biosystems).

### Western blot analysis of NS3 expression

MDA-MB-435-GFP and shDRBP76-cells were incubated with dengue virus in infection media for 1 hr at a MOI of 0.5. After the infection, the media containing the virus was removed and replaced by fresh infection media, followed by incubation for the indicated times at 37°C. Cells were washed three times with PBS and lysed in triton X-lysis buffer (20 mM Tris-HCl pH 7.5, 150 mM NaCl, 1 mM EDTA, 1% Triton X-100, 7 mM β-ME and a cocktail of protease inhibitors [Invitrogen]). NF90 was detected with a mouse monoclonal anti-NF90 antibody (DRBP76, BD Biosciences; 1∶250 dilution), and accumulation of dengue virus NS3 protein was detected using a rabbit polyclonal antibody (Dr. R. Padmanabhan, Georgetown University). As a loading control for all western blots, levels of NF90 and NS3 were normalized to β-actin levels, using a β-actin mouse monoclonal antibody (Abcam). Bands were visualized with the Western Lightning® Western Blot Chemiluminescence Reagent (Perkin Elmer), using the Alpha Innotech Imager 5500 (Alpha Innotech). Quantification was done using the AlphaEaseFC software (Alpha Innotech) followed by statistical analysis using Microsoft Excel.

### Real-time RT-PCR analysis

To prepare RNA for RT-PCR analysis, dengue infected cells were washed twice with PBS and then lysed with Trizol (Invitrogen) according to the manufacturer's instructions. Glycogen carrier (Glycoblue, Ambion) was added prior to alcohol precipitation to aid in visualizing the RNA pellets, which were resuspended in 20 µL of RNAse-free water. Complementary DNA was synthesized using SensiScript RT Kit (Qiagen) according to the manufacturer's instructions. Oligo-dT and random hexamers were used as primers at 1 mM and 10 mM final concentrations, respectively.

Real-time PCRs were carried out in triplicate, with each reaction using two µL of the previously described reverse transcription reaction as template. PCR was carried out in a final volume of ten µL, using the QuantiFast Probe PCR + Rox Vial 2x reagent (Qiagen). As a control, actin cDNA was amplified using the human β-actin primer/VIC-TAMRA probe mix (furnished at 20x, ABI). Dengue cDNA was amplified using primers D2F (5′-AAGGTGAGATGAAGCTGTAGTCTC-3′), D2R (5′-ATTCCATTTTCTGGCGTTCT-3′), and FAM-labeled probe D2P (5′-6FAM-CTGTCTCCTCAGCATCATTCCAGGCA-TAMRA-3′). Primers and probe were used at final concentrations of 0.9 mM and 0.25 mM, respectively. Reactions were carried out in an Opticon 2 (Bio-Rad) thermal cycler using 40 cycles of: 95°C, 15 sec; 60°C, 15 sec, 72°C, 30 sec. Thresholds were manually set to determine *C_T_* values for each sample using the Opticon Monitor 3 software (BioRad), and linear ranges of amplification were determined using LinRegPCR software [55]. The ratio of dengue RNA levels in shDRBP76-GFP cells to those in MDA-MB-435-GFP cells was determined by the ΔΔ*C_T_* method, using β-actin *C_T_* values for normalization. Statistical significance was tested using the randomization method described by Pfaffl *et al.* [26] performed by REST-2009 software (Qiagen).

### Flow cytometry

MDA-MB-435-GFP cells and shDRBP76-GFP cells were mock-infected or infected with dengue virus in triplicate wells, as described above. Culture medium was collected at 24, 36, and 48 hours post-infection. Media from triplicate wells were pooled and used to infect 105 K562 cells that express a putative dengue virus receptor, DC-SIGN [27], [56] for one hour at 37°C/5% CO2. Fourteen hours post-infection, cells were washed with PBS and fixed for 30 min at 4°C in PBS/4% paraformaldehyde/1% FBS. Cells were washed once with PBS/1% FBS and permeabilized with PBS/0.1% saponin/1% FBS for 30 min at 4°C, then washed twice with PBS and incubated with dengue virus prM monoclonal antibody (clone D3-2H2-9-21, Millipore) at 1∶200 in PBS/0.1% saponin/1% FBS for 30 min at 4°C. After three washes with PBS/1% FBS, cells were incubated with AlexaFluor 488-conjugated goat anti-mouse IgG at 1∶200 in PBS/0.1% saponin/1% FBS for 30 min at 4°C. Cells were washed twice with PBS/1% FBS and resuspended in PBS/4% paraformaldehyde/1% FBS. Flow cytometry was carried out with a BD LSR II instrument, with data analysis using FlowJo software (TreeStar).
